# Supplementary material for: Lithiophilic-lithiophobic gradient interfacial layer for a highly stable lithium metal anode
Source: Nat Commun. 2018 Sep 13;9:3729. doi: 10.1038/s41467-018-06126-z (PMC6137161; doi:10.1038/s41467-018-06126-z)
Supplement: Supplementary file 1 — Supplementary Information [file 41467_2018_6126_MOESM1_ESM.pdf]

Supplementary Information

Lithiophilic-lithiophobic gradient interfacial layer for highly stable lithium metal anode

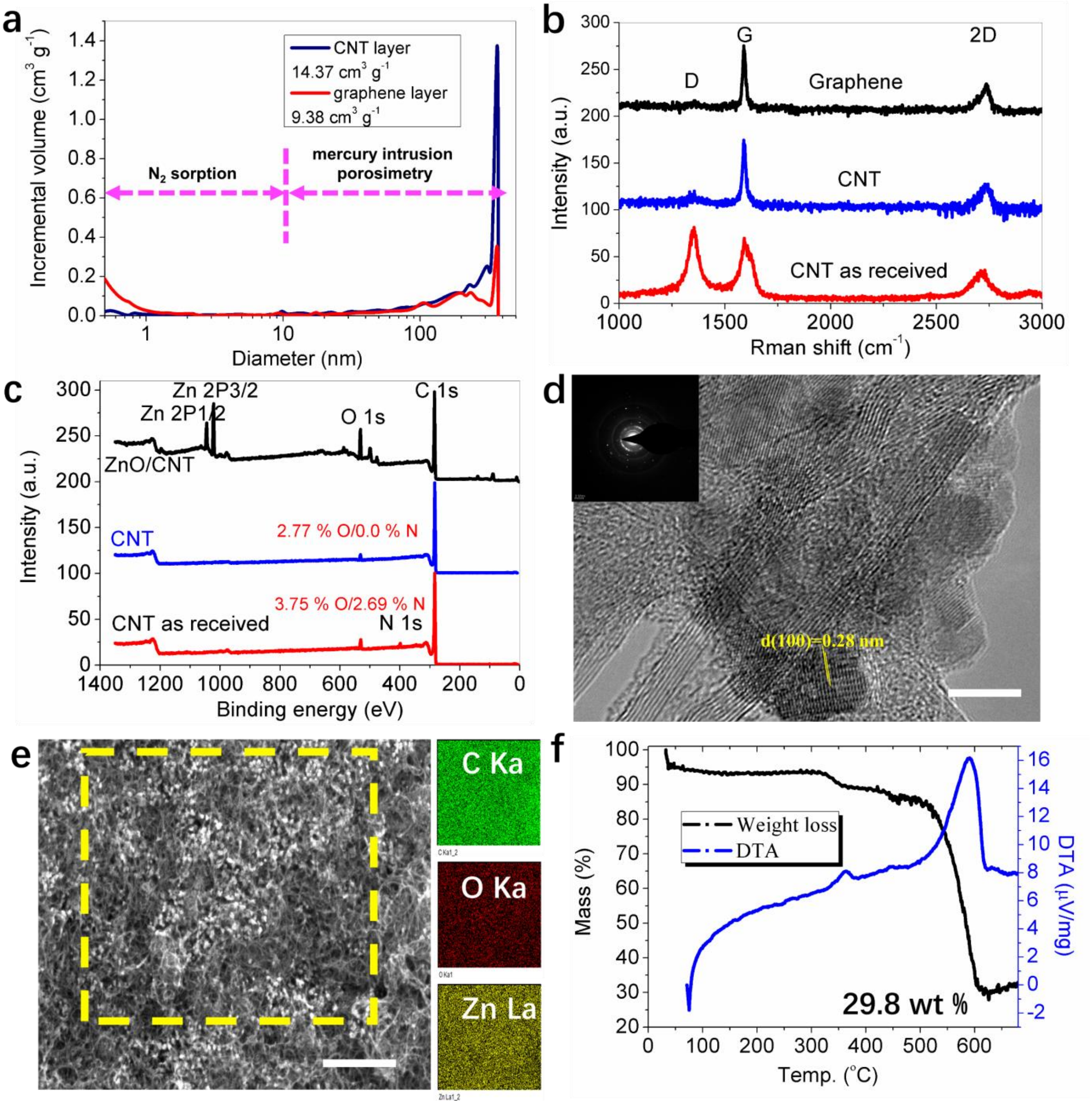

Supplementary Figure 1. Characterization of CNT and graphene. (a), Pore size distribution of CNT and graphene films by N_2 sorption and mercury intrusion porosimetry. (**b**) Raman spectra of the CNT as-received and after heat treatment, and graphene. (**c)** XPS of the CNT as-received and after heat treatment, and ZnO/CNT. (**d**) HRTEM image of ZnO/CNT. (**e**) SEM images of ZnO/CNT. Insets in **e** is the corresponding C, O, and Zn elemental EDX mapping image of ZnO/CNT highlighted by yellow**. (f**) Thermogravimetric curve in air atmosphere of ZnO/CNT. Scale bar, 5nm for (**d**) and 500μm for (**e**).

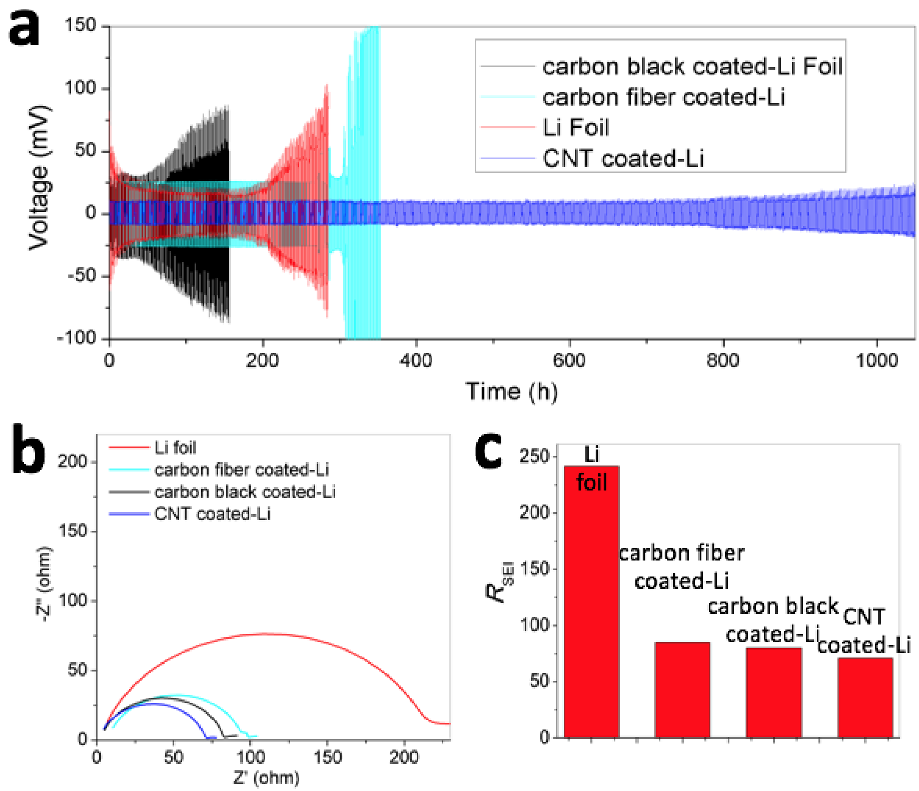

Supplementary Figure 2. Electrochemical performance of blank Li foil cell and cells assembled by Li foils with carbon fiber, carbon black, and CNT interfacial layers. (a), Comparison of the cycling stability of a symmetric cell assembled by blank Li foils (red), and Li foils with interfacial layers of carbon fiber (light blue), carbon black(black), and CNT (blue), respectively, at charge/discharge current densities of 1 mA cm^{-2} with a stripping/plating capacity of 1 mAh cm^{-2} for as long as 520 cycles. (**b**) Electrochemical impedance spectra of various cells. Frequency: 0.1 Hz–1 MHz, perturbation amplitude 5 mV, measured at 0% state of charge (SOC). (**c**) Summary of R_{SEI} fitting results of all cells.

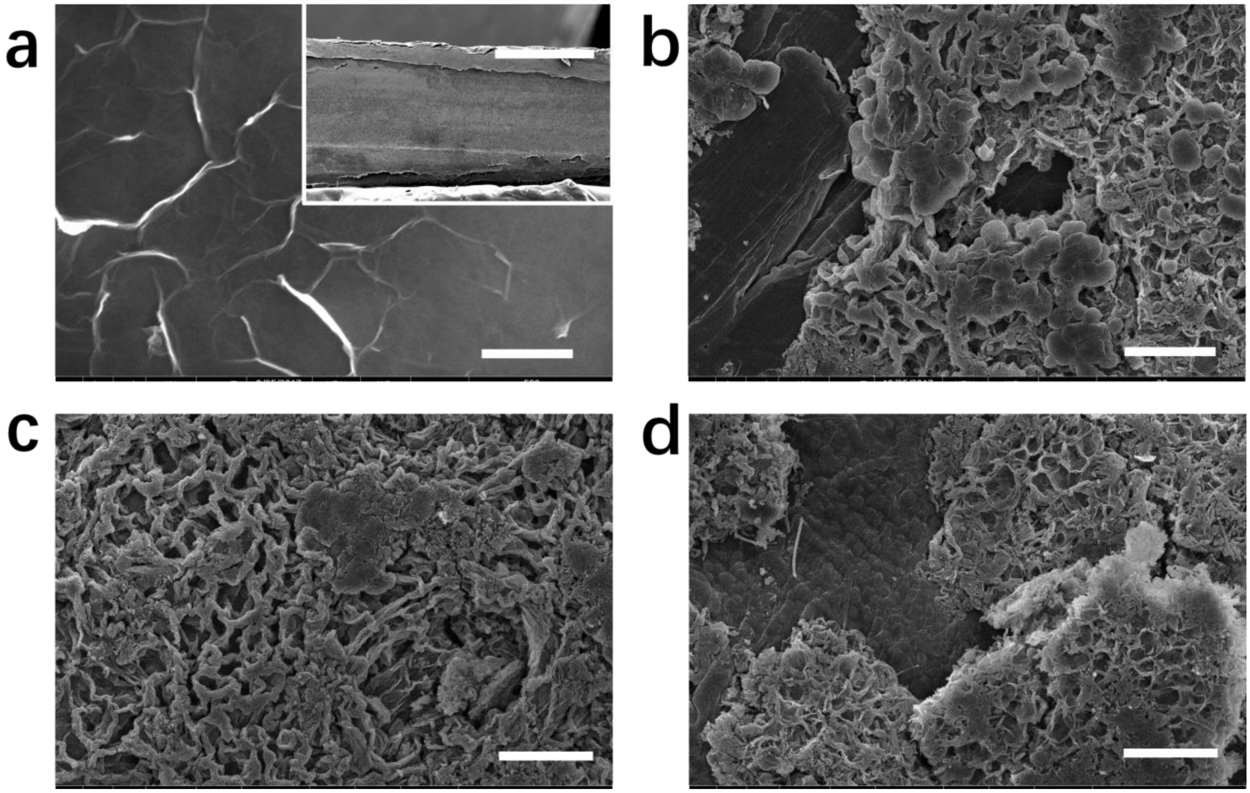

Supplementary Figure 3. SEM images of top view morphology of the graphene interfacial layer coated Li foils. (a) Pristine graphene coated Li foil. Inset is the cross section view of the graphene coated Li foil. Scale bar, 500nm for (**a**) and 200μm. (**b-d**) SEM image with various magnifications of graphene coated Li symmetric cells cycled at a charge/discharge current density of 1 mA cm^{-2} with a stripping/plating capacity of 1 mAh cm^{-2} after 210 cycles. Scale bar, 20μm for (**b-d**).

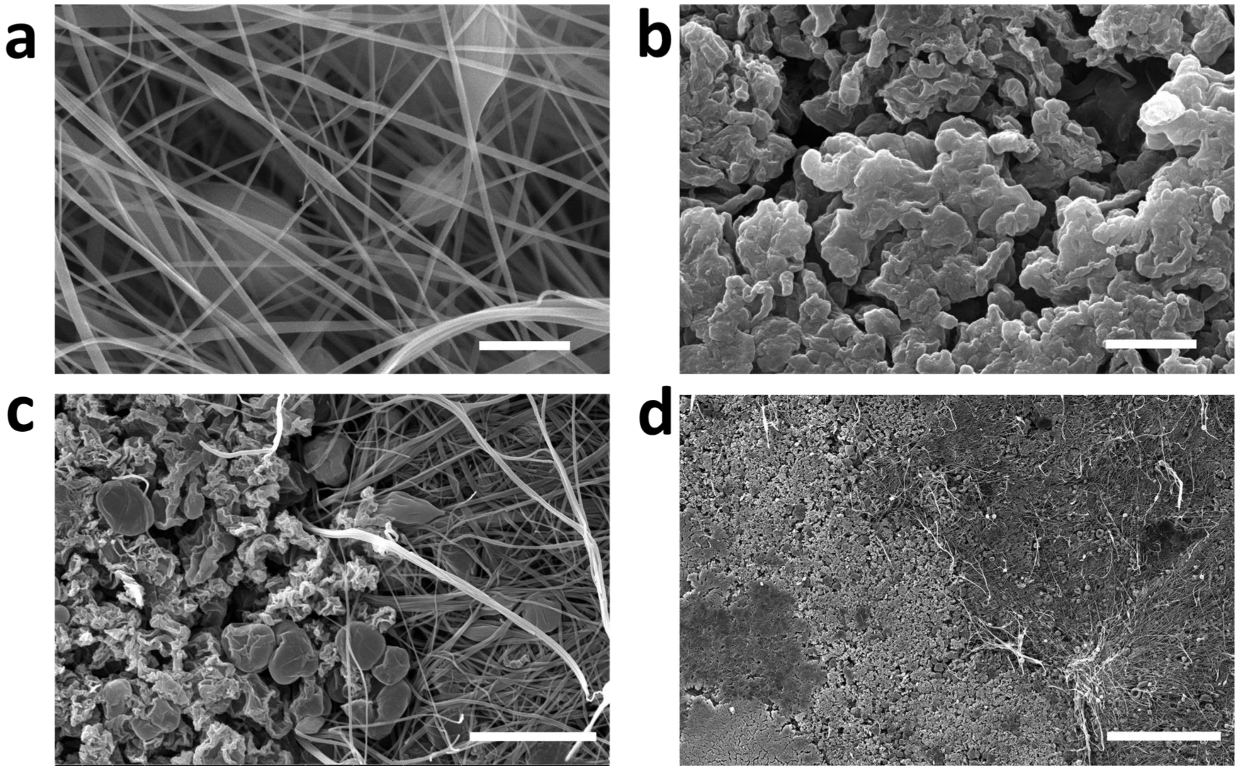

Supplementary Figure 4. SEM images of top view morphology of the electrospun fiber interfacial layer coated Li foils. (a) Pristine electrospun fiber coated Li foil. (**b-d**) SEM image with various magnifications of electrospun fiber coated Li symmetric cells cycled at a charge/discharge current density of 1 mA cm^{-2} with a stripping/plating capacity of 1 mAh cm^{-2} after 180 cycles. Scale bar, 1μm, 2μm, 5μm, 50μm for (**a-d**).

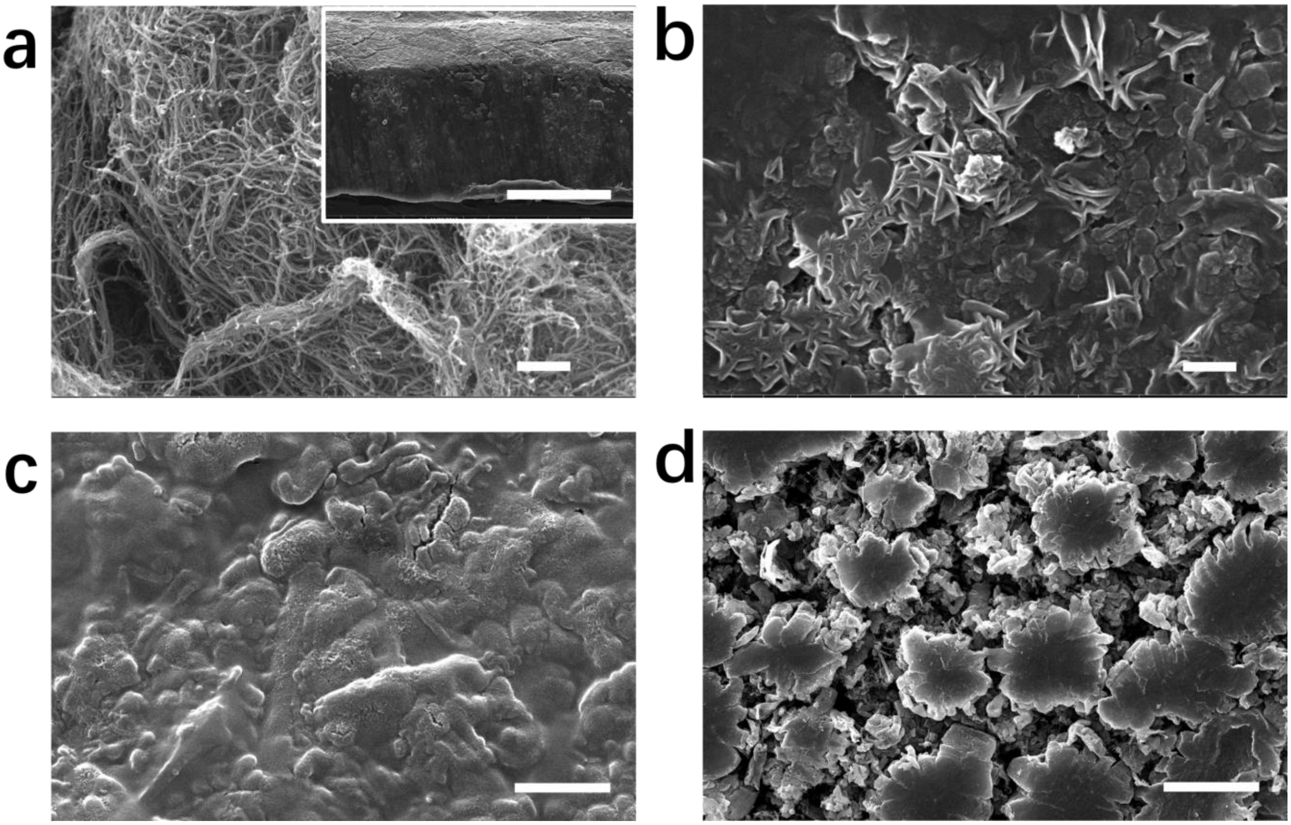

Supplementary Figure 5. SEM images of top view morphology of the ZnO/CNT interfacial layer coated Li foils. (a) Pristine ZnO/CNT coated Li foil. Inset is the cross section view of the ZnO/CNT coated Li foil. (**b-d)**, SEM image with various magnifications of ZnO/CNT coated Li symmetric cells cycled at a charge/discharge current density of 1 mA cm^{-2} with a stripping/plating capacity of 1 mAh cm^{-2} after 280 cycles. Scale bar, 500nm, 500nm, 1μm, 20μm for (**a-d**) and 200μm for the inset of (**a**).

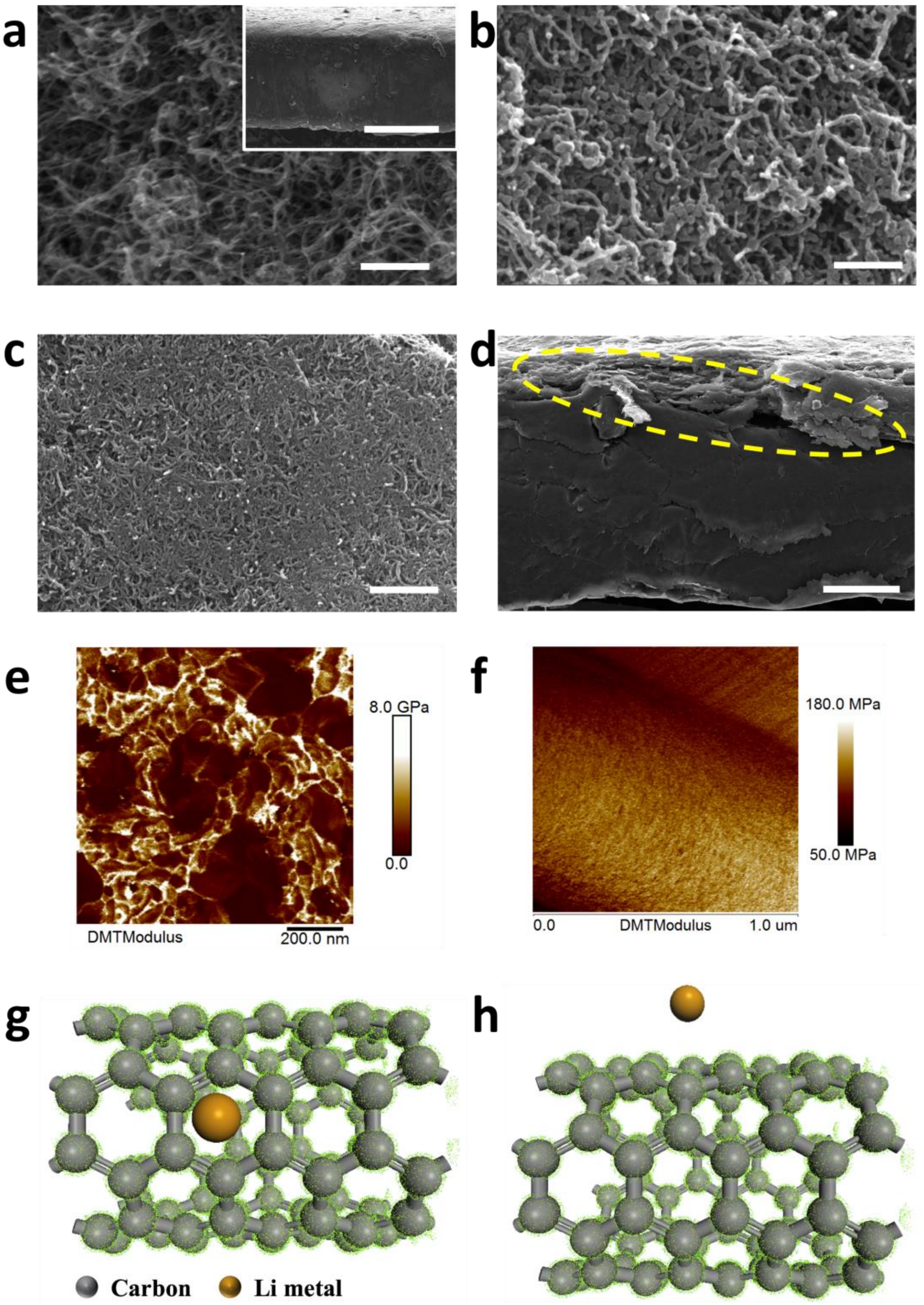

Supplementary Figure 6. SEM images of top view morphology of the CNT interfacial layer coated Li foils. (a) As prepared CNT coated Li foil. Inset is the cross section view of the CNT coated Li foil.

(**b-c**) SEM image with various magnifications of CNT coated Li symmetric cells cycled at a charge/discharge current density of 1 mA cm^{-2} with a stripping/plating capacity of 1 mAh cm^{-2} after 520 cycles. (**d**) Cross section view of the CNT coated Li foil at charge/discharge current densities of 5 mA cm^{-2} with a stripping/plating capacity of 1 mAh cm^{-2} for 220 cycles, with the gap between CNT layer and Li foil marked by yellow dash lines. **e**, **f**, AFM Young's modulus mapping of the CNT **(e)** and electrospun fiber **(f)** interfacial layers. (**g**) Top view and (**h**) cross section view of Li atom binding with carbon atom of CNT calculated with DFT using the CASTEP code in Materials Studio (version 2017 R2) of Accelrys Inc. The local potential of CNT is shown as green dots. Scale bar, 500nm, 500nm, 1μm, 100μm for (**a-d**) and 200μm for the inset of (**a**).

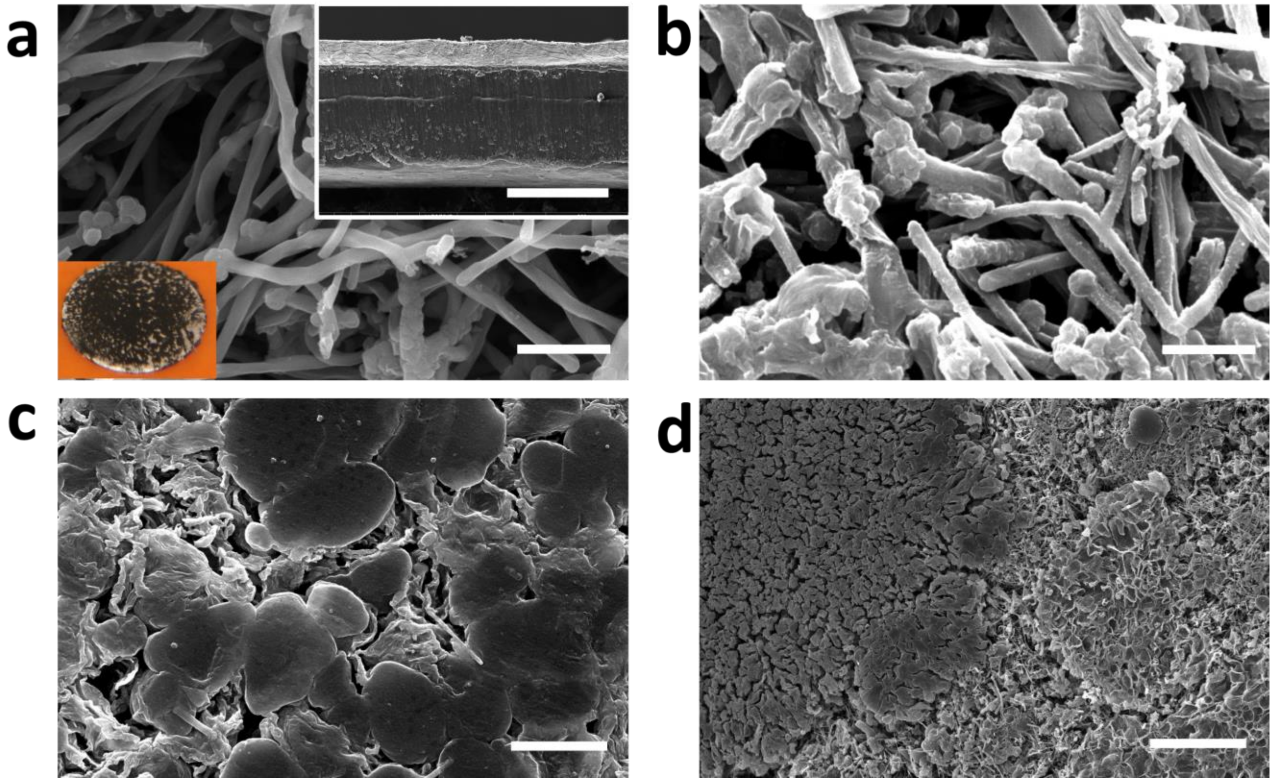

Supplementary Figure 7. SEM images of top view morphology of the carbon fiber interfacial layer coated Li foils. (a) Pristine carbon fiber coated Li foil. Inset is the cross section view of the carbon fiber coated Li foil. The under right inset show the digital photos of the corresponding Li foils with carbon fiber interfacial layers, indicating the bad film-forming property of carbon fiber. (**b-d**) SEM image with various magnifications of carbon fiber coated Li symmetric cells cycled at a charge/discharge current density of 1 mA cm^{-2} with a stripping/plating capacity of 1 mAh cm^{-2} after 170 cycles. Scale bar, 1μm, 1μm, 5μm, 20μm for (**a-d**) and 200μm for the inset of (**a**).

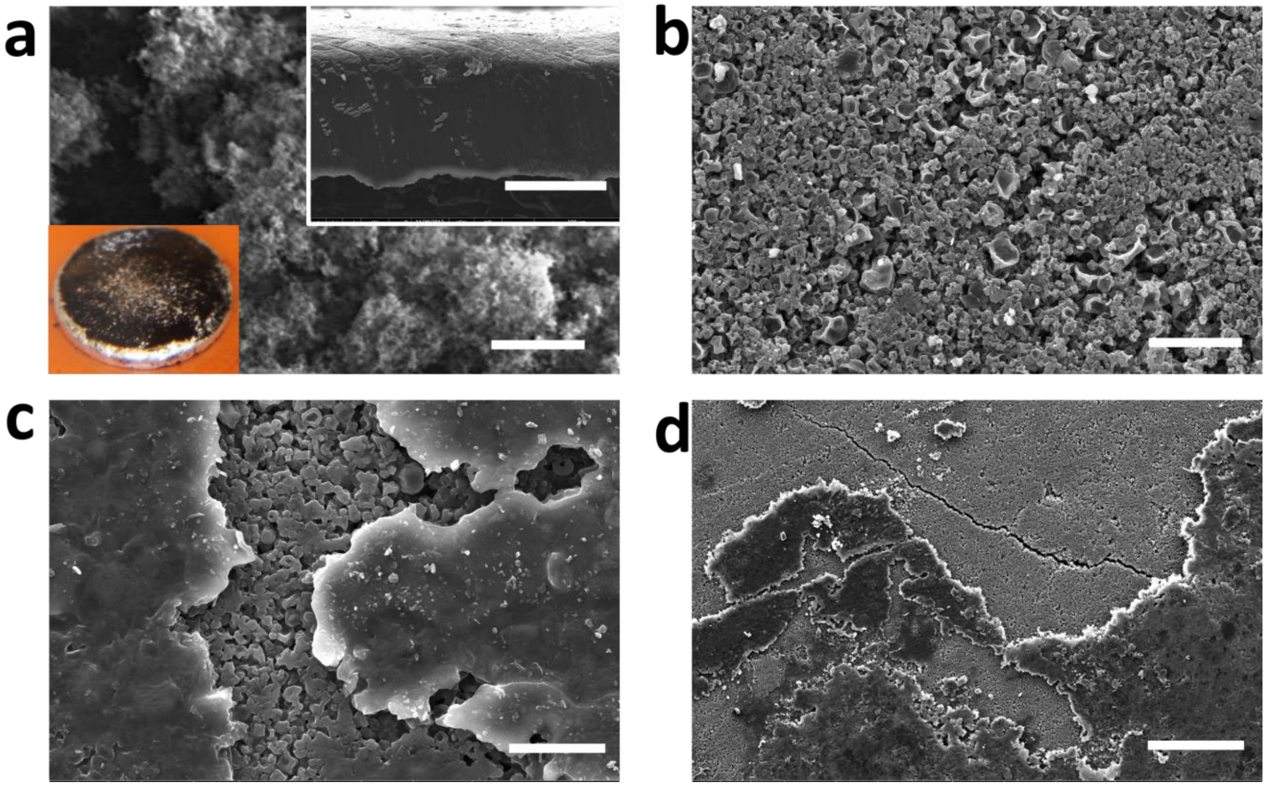

Supplementary Figure 8. SEM images of top view morphology of the carbon black interfacial layer coated Li foils. (a) Pristine carbon black coated Li foil. Inset is the cross section view of the carbon black coated Li foil. The under right inset show the digital photos of the corresponding Li foils with carbon black interfacial layers, indicating the bad film-forming property of carbon black. (**b-d**) SEM image with various magnifications of carbon black coated Li symmetric cells cycled at a charge/discharge current density of 1 mA cm^{-2} with a stripping/plating capacity of 1 mAh cm^{-2} after 140 cycles. Scale bar, 500nm, 20μm, 20μm, 200μm for (**a-d**) and 200μm for the inset of (**a**).

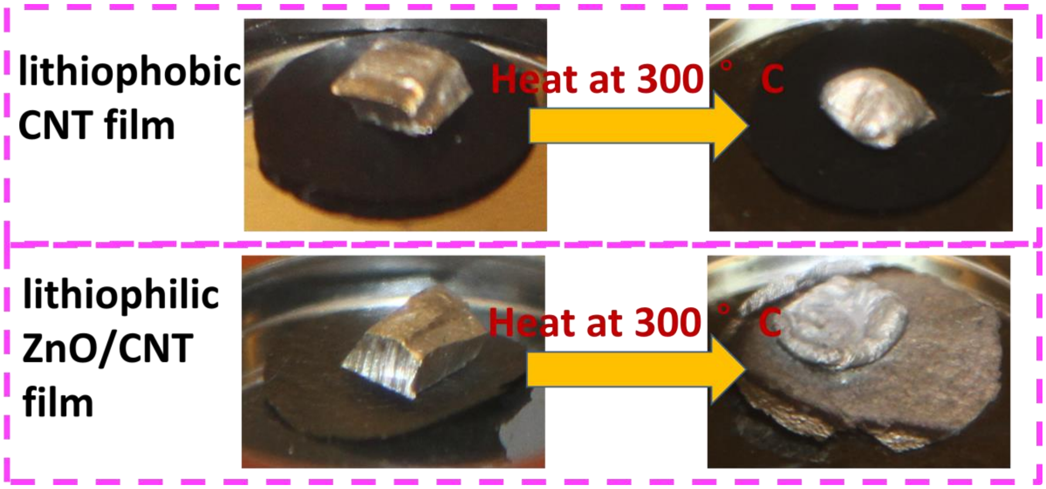

Supplementary Figure 9. Characterization of the lithiophobic and lithiophilic properties of CNT and ZnO/CNT. Li wetting property of CNT films without (upper) and with (under) ZnO coating.

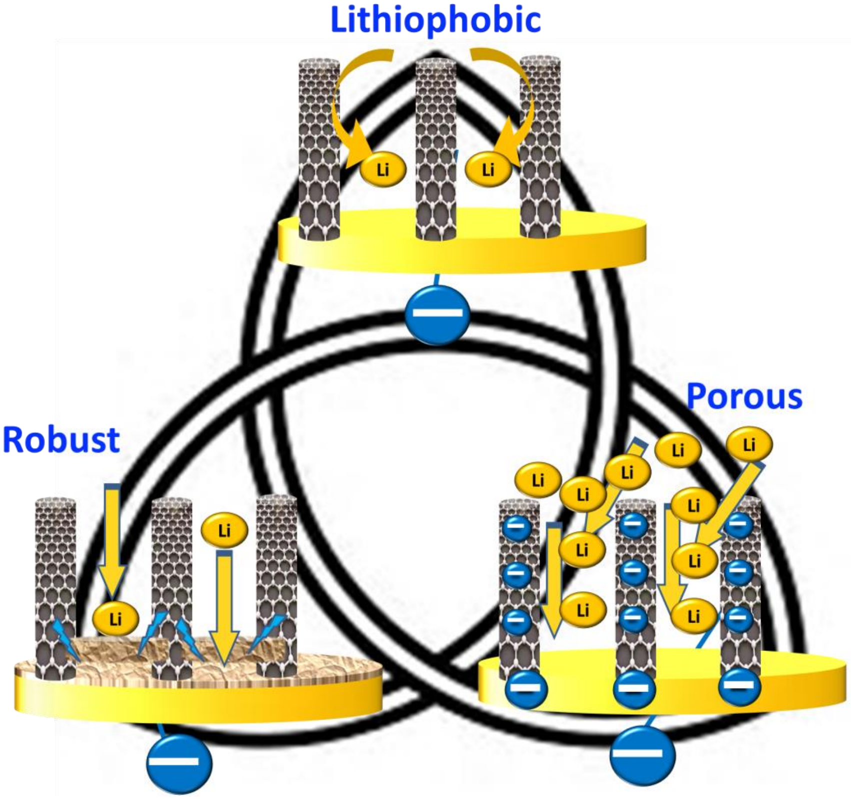

Supplementary Figure 10. Schematic of prerequisites for an ideal Li foil interfacial layer. Interfacial layer should be lithiophobic and robust to avoid the penetration of Li dendrite, while it should also be porous to ensure the fast diffusion of Li ions through it.

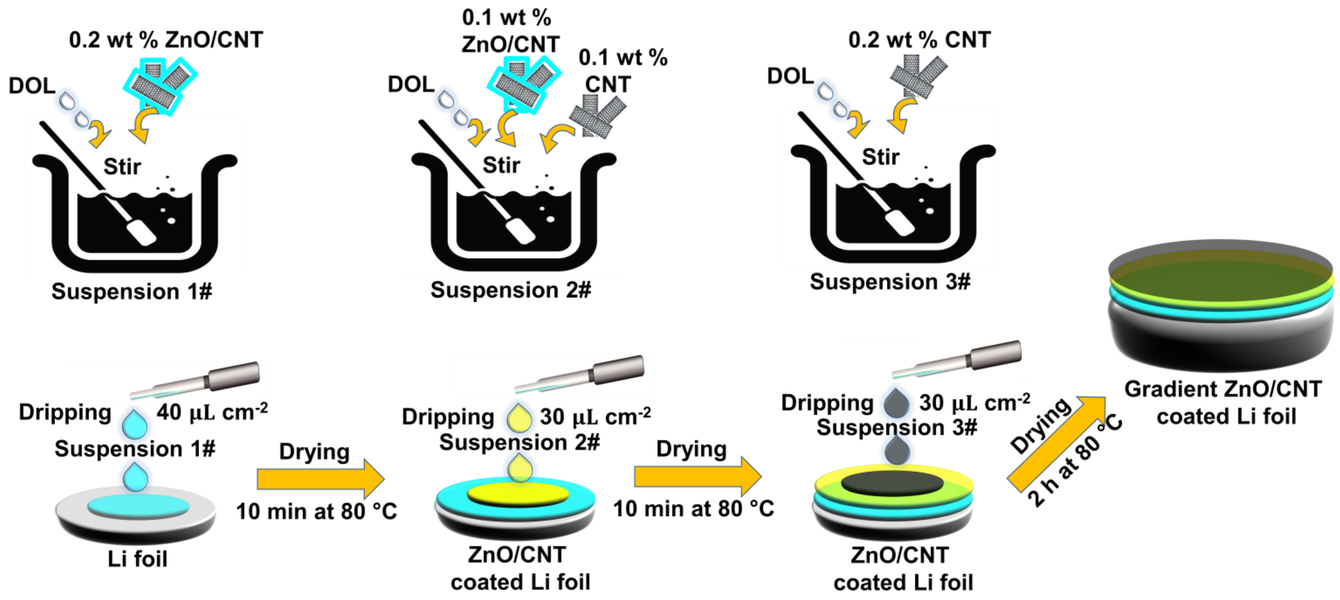

Supplementary Figure 11. Schematic of lithiophilic/lithiophobic gradient interfacial layer fabrication in glove box. 0.2 wt. % ZnO/CNT and 0.2 wt. % CNT was mixed with 1,3-dioxolane (DOL), and the suspension were marked as 1# and 3#, respectively. Suspension 1# was mixed with suspension 2# with a volumetric ratio of 1:1 as suspension 2#. Suspension 1#, 2#, and 3# was subsequently dripped onto the Li foil by a mass loading of 40. 30, and 30 μL cm^{-2}, with a drying for 10 min at 80 °C after each dripping and a final drying for 2 h at 80 °C to obtain the gradient ZnO/CNT interfacial layer.

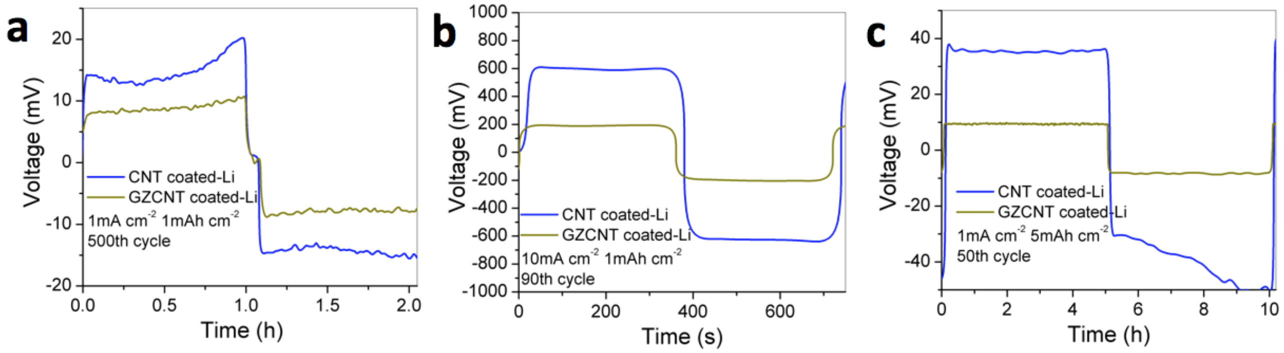

Supplementary Figure 12. Electrochemical performance of cells assembled by Li foils with GZCNT interfacial layers. a-c, Typical Li stripping/plating profiles of symmetric cell with CNT (blue) and GZCNT (brown) coated Li foils, at charge/discharge current densities of (**a**) 1 and (**b**) 10 mA cm^{-2} with a stripping/plating capacity of 1 mAh cm^{-2} of the (**a**) 500th and (**b**) 90th cycles, and (**c**) at 1 mA cm^{-2} with 5 mAh cm^{-2} stripping/plating capacity of the 50th cycle.

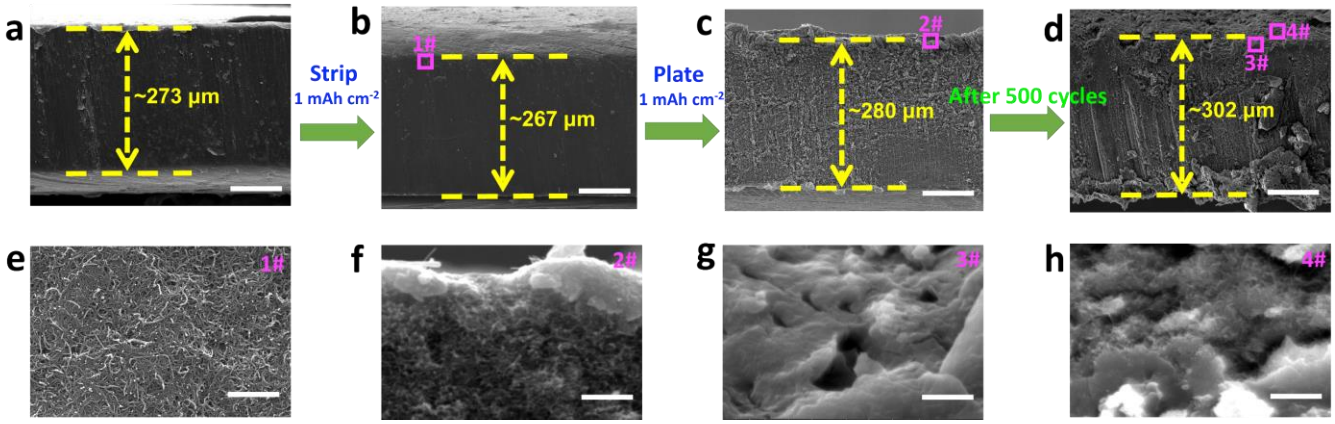

Supplementary Figure 13. Well-confined stripping/plating behavior of the GZCNT coated Li foil. a-h, SEM images of cross-section view morphology of **(a)** pristine GZCNT coated Li foil and **(b)** after striping 1 mAh cm^{-2}, **(c)** after plating back 1 mAh cm^{-2}, and **(d)** after 500 cycles at 1 mA cm^{-2}. (**e-h**) Corresponding SEM images highlighted by pink in b-d, confirming that the morphology of GZCNT layer is well kept from initial stripping/plating to after-500-cycle and no Li dendrite is visible. Scale bar, 100μm for (**a-d**) and 1μm for (**e-h**).

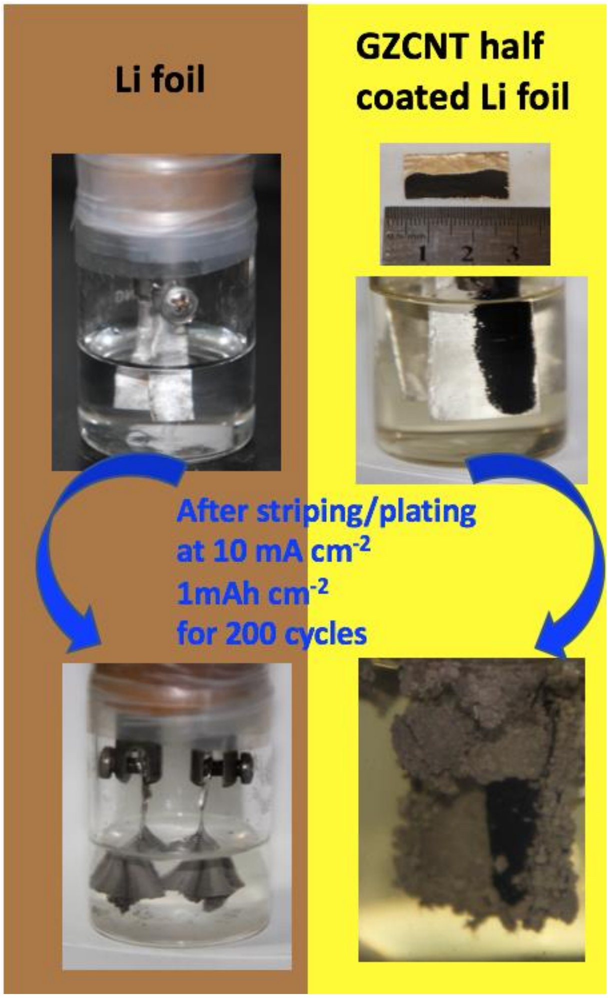

Supplementary Figure 14. In-situ observation of blank and GZCNT coated Li foil before and after Li stripping/plating. Li foil is half coated by GZCNT layer to clearly show its protection of Li anode from the dendrite formation. The electrolyte employed was 0.6 M lithium bis(tri-fluoromethanesulfonyl)imide and 0.4 M $LiNO_3$ in 1:1 w/w DOL/DME. The cells are charge/discharge at 10 mA cm^{-2} with 1mAh cm^{-2} capacity for 200 cycles.

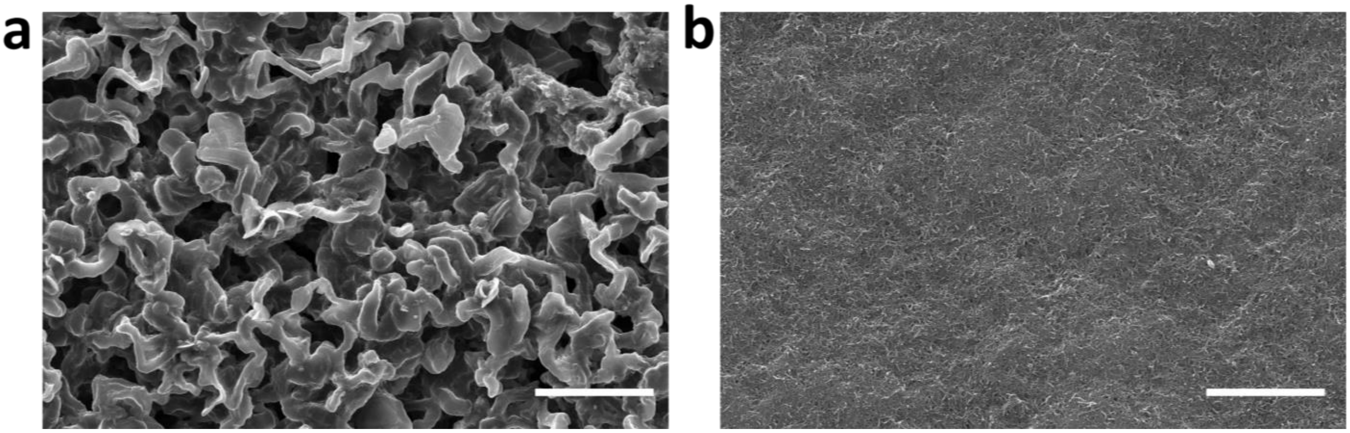

Supplementary Figure 15. SEM images of top view morphology of the (a) pristine Li foil and (b) GZCNT coated Li foil in punch cells cycled at charge/discharge current density of 1 mA cm^{-2} (10 mA in total) with a stripping/plating capacity of 1 mAh cm^{-2} (10 mAh in total) after 100 and 200 cycles. Scale bar, 50μm for (**a**) and 5μm for (**b**).

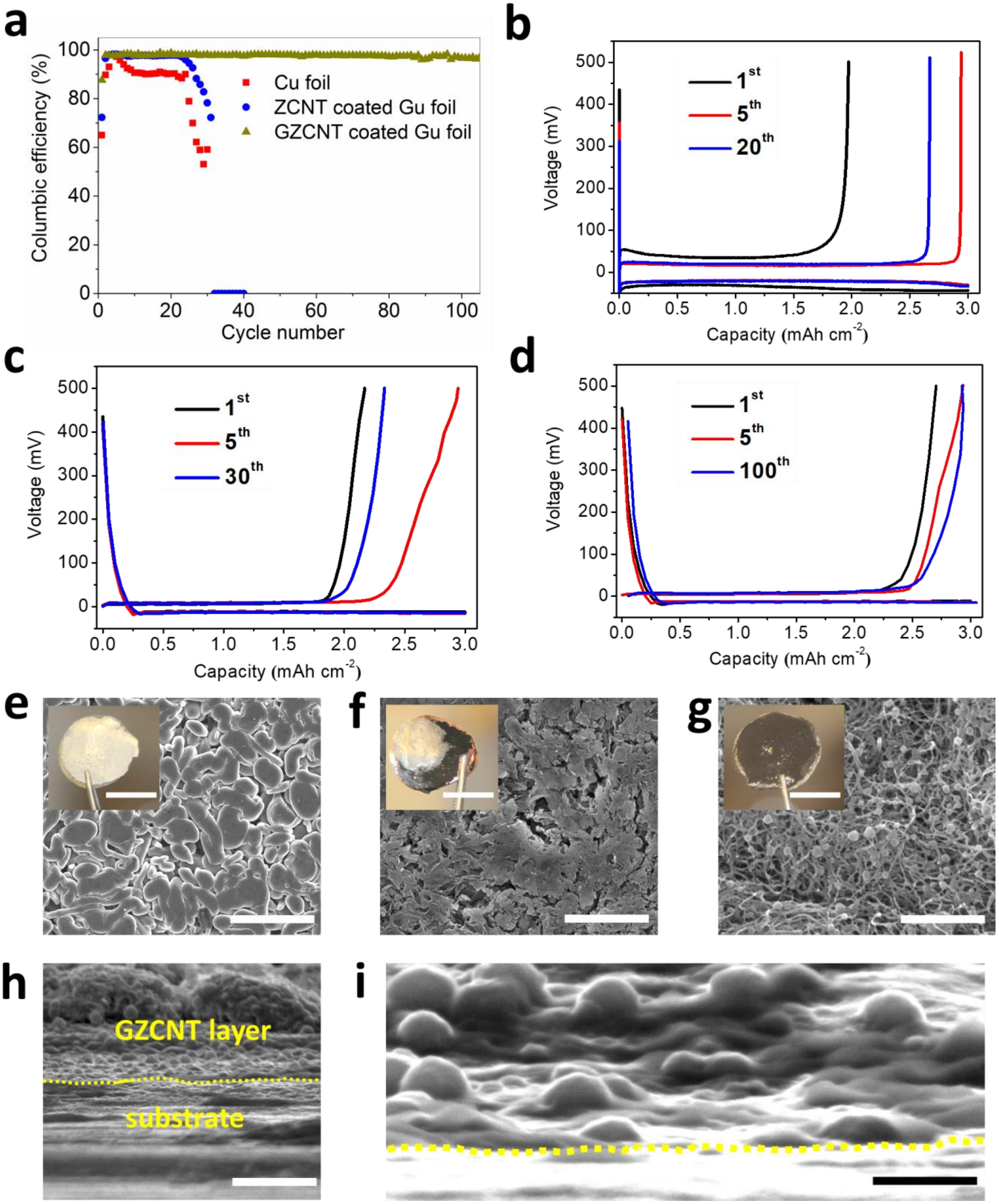
a
Columbic efficiency (%)
Cycle number
Cu foil
ZCNT coated Gu foil
GZCNT coated Gu foil
b
Voltage (mV)
Capacity (mAh cm-2)
1st
5th
20th
c
Voltage (mV)
Capacity (mAh cm-2)
1st
5th
30th
d
Voltage (mV)
Capacity (mAh cm-2)
1st
5th
100th
e
f
g
h
GZCNT layer
substrate
i

Supplementary Figure 16. The characterization of Cu|Li asymmetrical cells with pristine, ZCNT and GZCNT coated Cu electrodes. (**a**) Comparison of cycling stability of pristine Cu electrodes (red) and ZCNT (blue) and GZCNT coated Cu electrodes (dark yellow) at current density of 2.0 mA cm^{-2} with a capacity of 3 mA h cm^{-2}. Voltage profiles of the Li plating/stripping process on (**b**) bare Cu electrodes, (**c**) ZCNT and (**d**) GZCNT coated Cu electrodes with a capacity of 3 mA h cm^{-2} at a current density of 2 mA cm^{-2}. SEM images of top view morphology of bare Cu electrode (**e**), ZCNT (**f**) and GZCNT coated Cu electrodes (**g**) after (**e**) 30, (**f**) 40, and (**g**) 110 cycles at 2 mA cm^{-2} with 3 mAh cm^{-2} stripping/plating capacity. The insets show the digital photos of the corresponding Cu current collectors with interfacial layers after test. The cross-section view morphology of GZCNT coated Cu electrode at low (**h**) and high (**i**) magnifications, showing a smooth surface with well-preserved thin layer of SEI. Scale bar, 20μm, 20μm, 2μm, 2μm, 500nm for (**e-i**) and 1cm for the inset of (**e-g**).

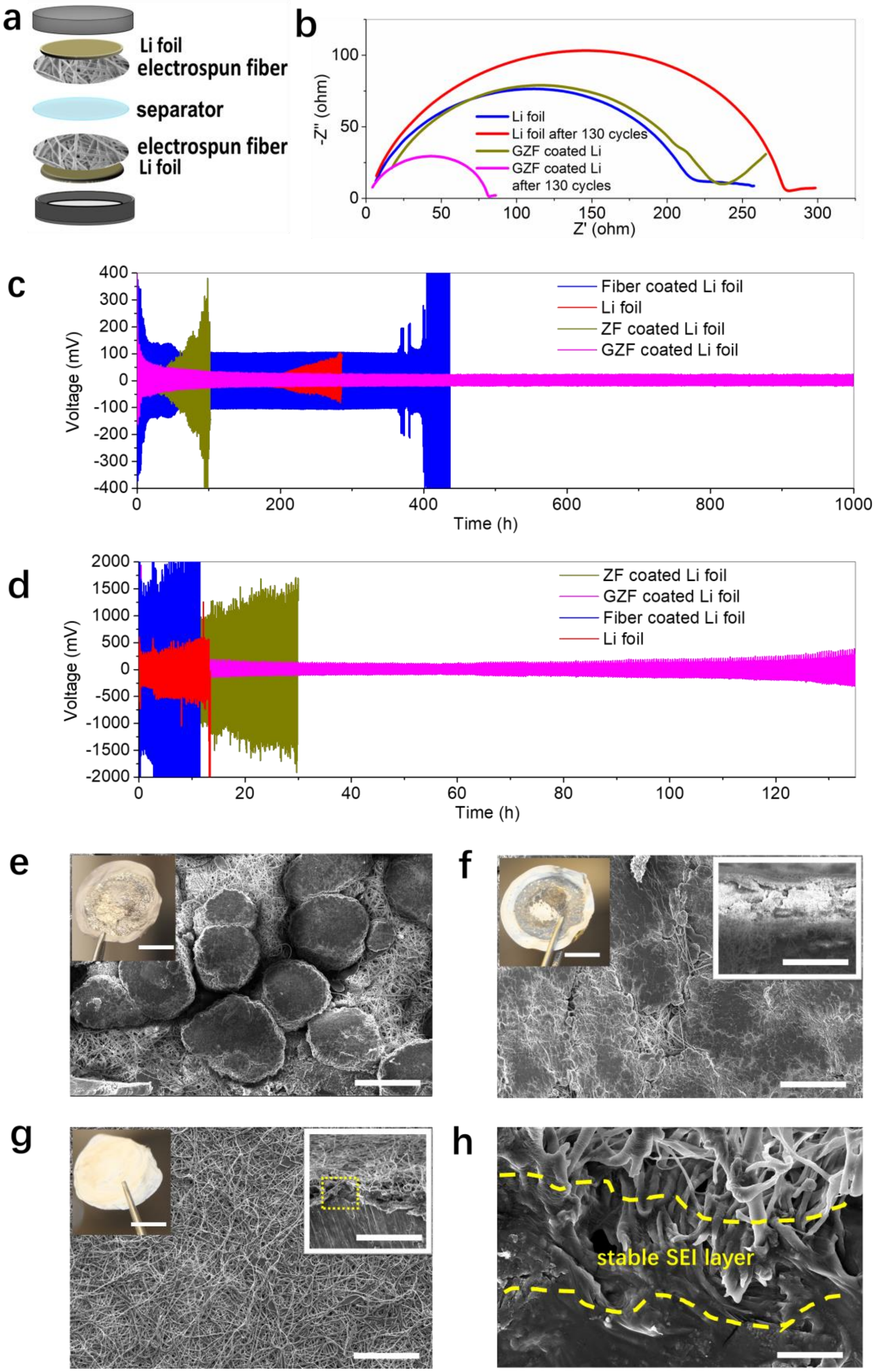
a
Li foil
electrospun fiber
separator
electrospun fiber
Li foil
b
-Z'' (ohm)
Z' (ohm)
Li foil
Li foil after 130 cycles
GZF coated Li
GZF coated Li
after 130 cycles
c
Voltage (mV)
Time (h)
Fiber coated Li foil
Li foil
ZF coated Li foil
GZF coated Li foil
d
Voltage (mV)
Time (h)
ZF coated Li foil
GZF coated Li foil
Fiber coated Li foil
Li foil
e
f
g
h
stable SEI layer

Supplementary Figure 17. Symmetrical cell testing of bare Li electrode and Li foils with various fiber-based interfacial layers. (**a**) Schematics of the cell design. **(b**) Electrochemical impedance spectra of various cells. Frequency: 0.1 Hz–1 MHz, perturbation amplitude 5 mV, measured at 0% state of charge (SOC). **c-d**, Comparison of the cycling stability of a symmetric cell assembled by blank Li foils (red), and Li foils with interfacial layers of electrospun fiber (blue), ZF (green), and GZF (dark yellow), respectively, at charge/discharge current densities of (**c**) 1 and (**d**) 5 mA cm^{-2} with a stripping/plating capacity of both 1 mAh cm^{-2}. SEM images of top view morphology of electrospun fiber (**e**), ZF (**f**) and GZF coated Li (**g**) after (**e**) 220, (**f**) 50, and (**g**) 500 cycles at 1 mA cm^{-2} with 1 mAh cm^{-2} stripping/plating capacity. The insets show the digital photos of the corresponding Li foils with interfacial layers after test. (**h**) Cross-section view morphology of GZF coated Li foil at high magnifications, showing a smooth surface with well-preserved stable layer of SEI. Scale bar, 20μm, 100μm, 100μm, 100μm, 10μm for (**e-h**), 1cm for the top left inset of (**e-g**) and 200μm, 100μm for top right of (**f-g**).

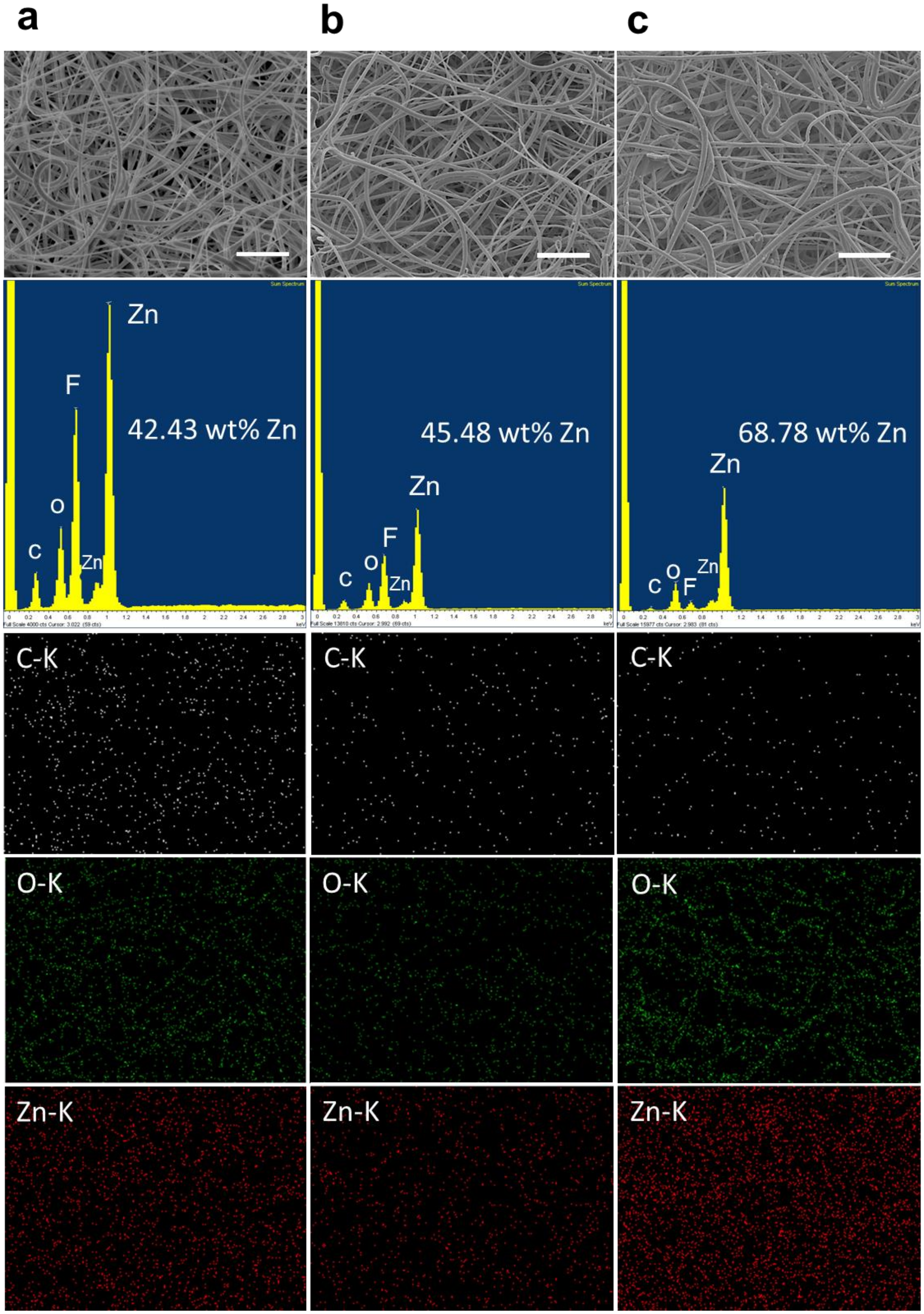
a
b
c
Zn
F
42.43 wt% Zn
O
C
Zn
45.48 wt% Zn
Zn
F
O
C
Zn
68.78 wt% Zn
Zn
C
O
F
Zn
C-K
C-K
C-K
O-K
O-K
O-K
Zn-K
Zn-K
Zn-K

Supplementary Figure 18. Characterization of the gradient ZnO-coated electrospun fiber (GZF) via magnetron sputtering. SEM images of the electrospun PVDF fibers from front (**a-c**) with different magnetron sputtering of (**a**) 40 min, (**b**) 120 min, (**c**) 240 min, and corresponding elemental EDX spectrum of GZF interfacial layer highlighted by yellow with elemental mapping of C, O and Zn distribution. Scale bar, 20µm for (**a-c**).

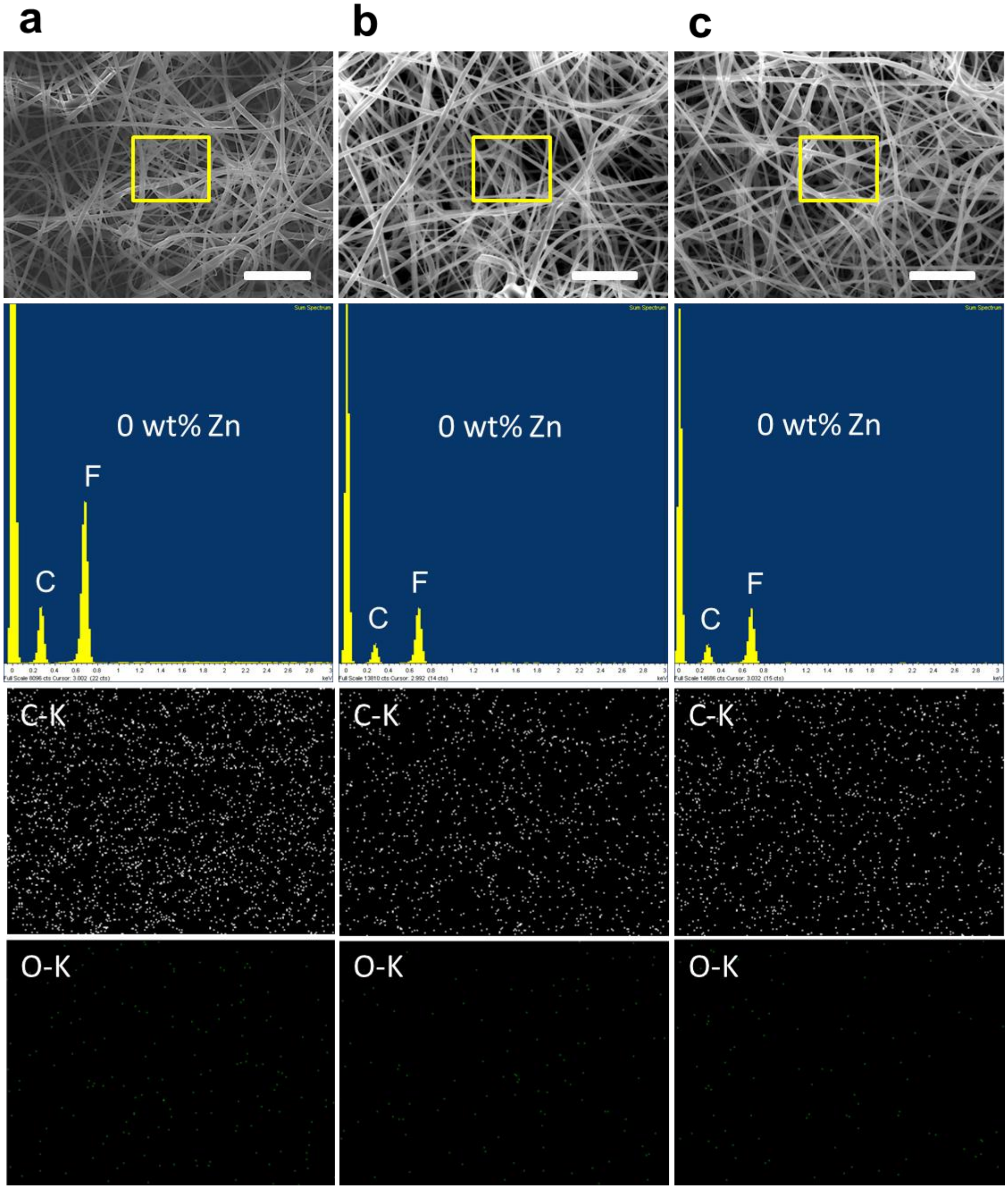

Supplementary Figure 19. Characterization of the gradient ZnO-coated electrospun fiber (GZF) via magnetron sputtering. SEM images of the electrospun PVDF fibers from back (**a-c**) view with different magnetron sputtering of (**a**) 40 min, (**b**) 120 min, (**c**) 240 min, and corresponding elemental EDX spectrum of GZF interfacial layer highlighted by yellow with elemental mapping of C, O and Zn distribution. Scale bar, 20μm for (**a-c**).

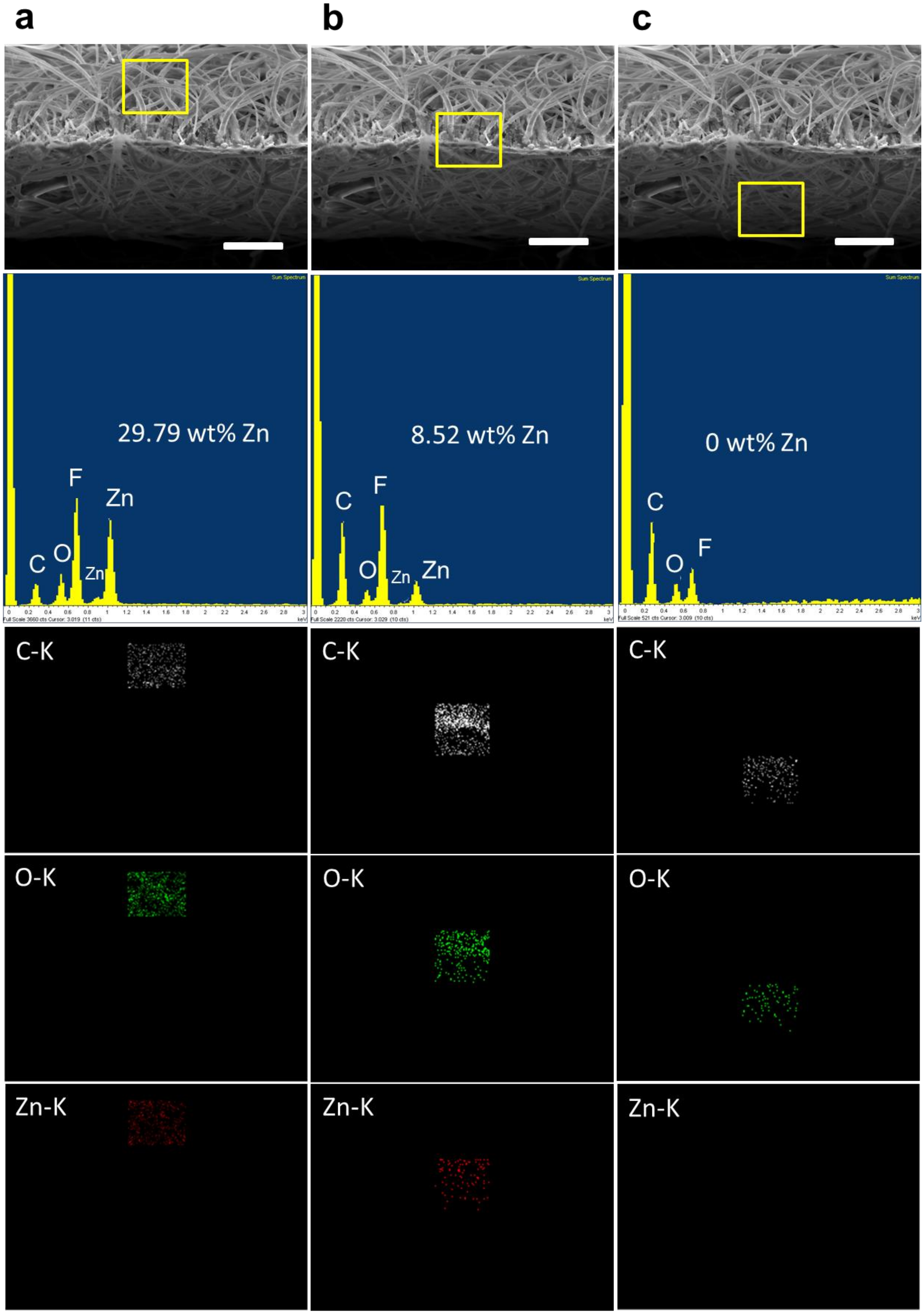
a
b
c
Sum Spectrum
29.79 wt% Zn
8.52 wt% Zn
0 wt% Zn
C
O
F
Zn
keV
C-K
O-K
Zn-K

Supplementary Figure 20. Characterization of the gradient ZnO-coated electrospun fiber (GZF) via magnetron sputtering. (**a-c**) Cross-section view morphology of the electrospun PVDF fibers with magnetron sputtering of 40 min, and corresponding elemental EDX spectrum of GZF interfacial layer from (**a**) front, (**b**) edge, (c) interior of SEM highlighted by yellow with elemental mapping of C, O and Zn distribution. Scale bar, 20μm for (**a-c**).

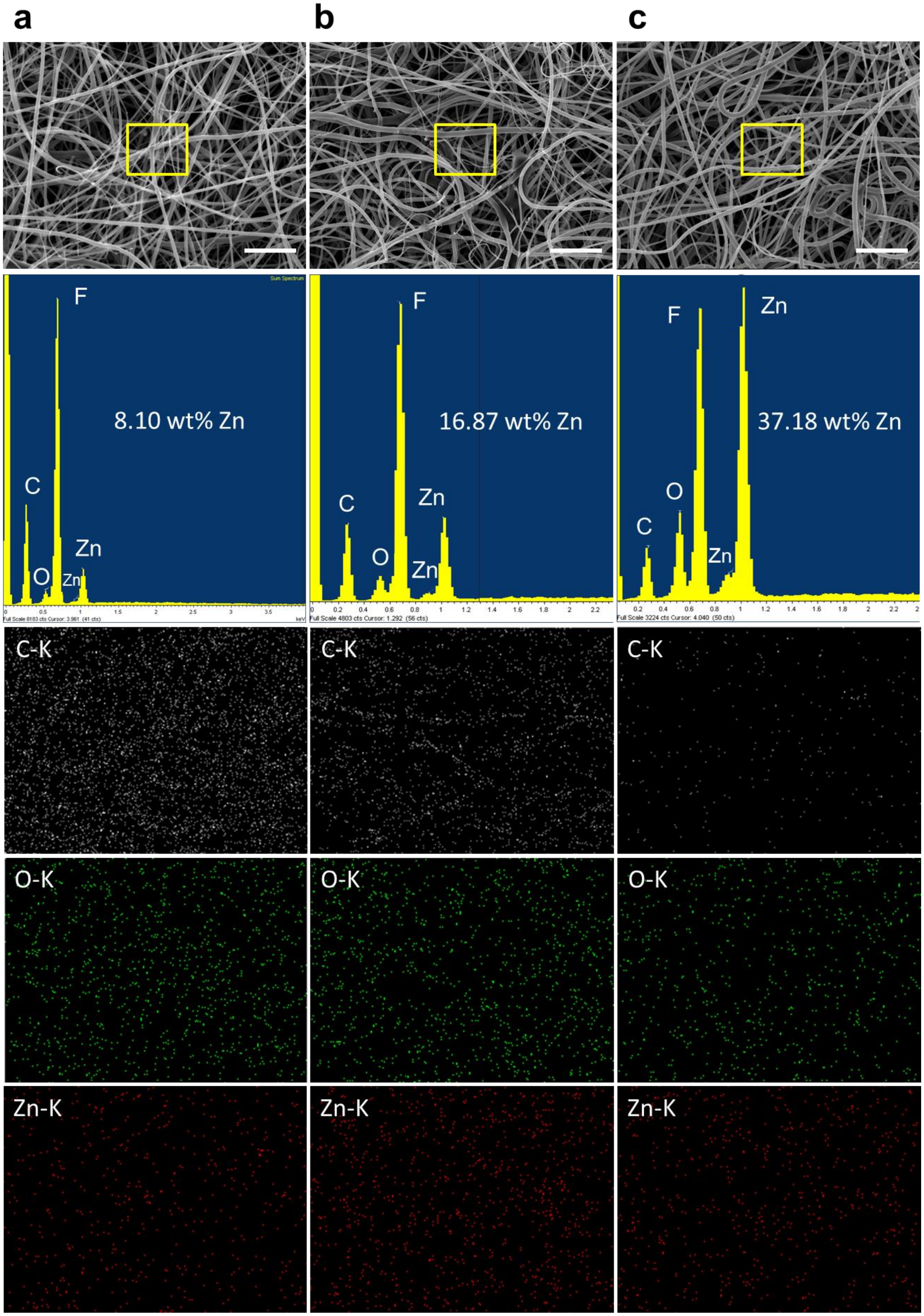

Supplementary Figure 21. Characterization of the ZnO-coated electrospun fiber (ZF) via ALD.

SEM images of the electrospun PVDF fibres from front (**a-c**) view with different coating thickness (**a**) 10 nm, (**b**) 20 nm, (**c**) 50 nm, and corresponding elemental EDX spectrum of GZF interfacial layer highlighted by yellow with elemental mapping of C, O and Zn distribution. Scale bar, 20μm for (**a-c**).

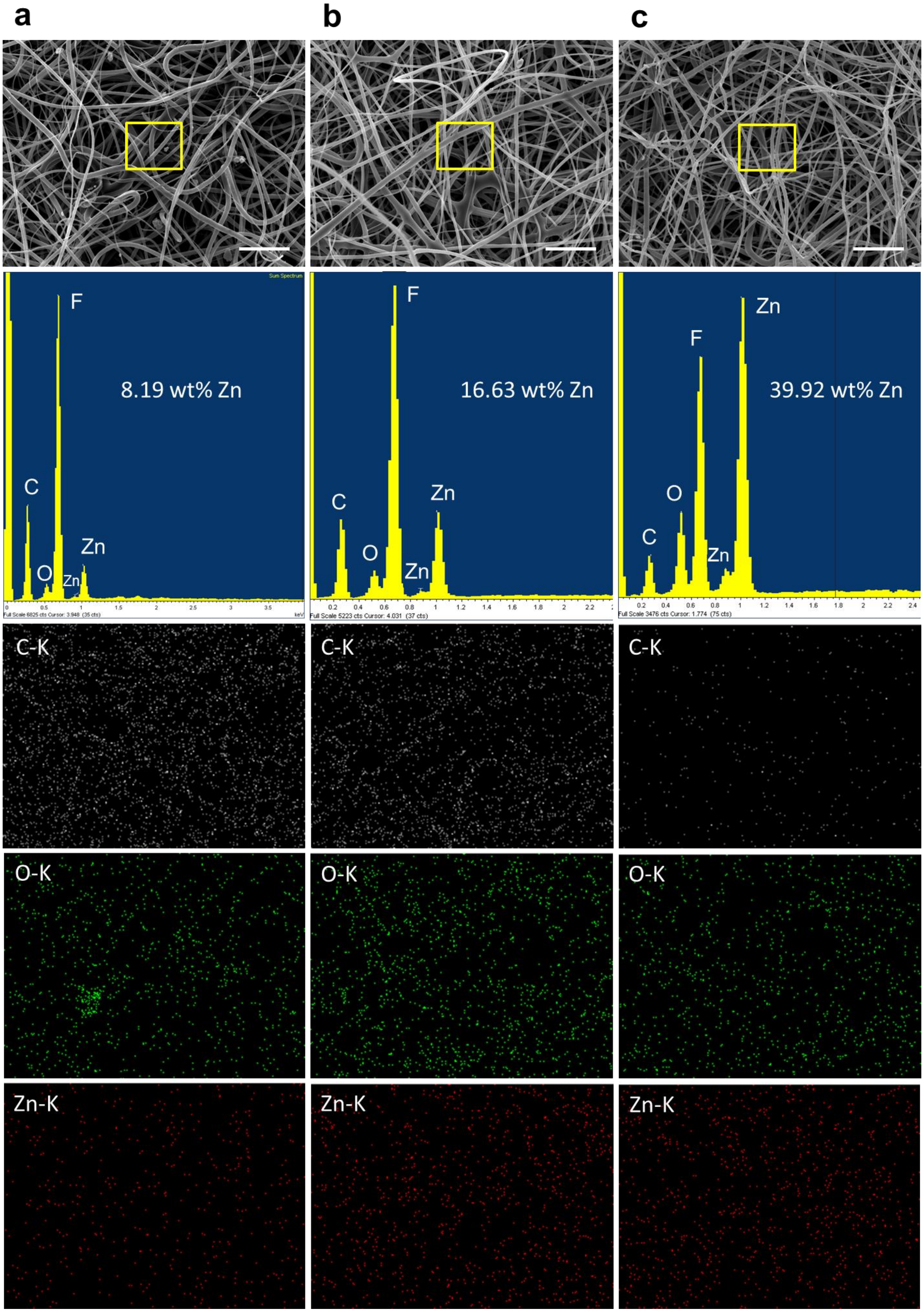
a
b
c
F
8.19 wt% Zn
C
O
Zn
Zn
16.63 wt% Zn
39.92 wt% Zn
C-K
O-K
Zn-K

Supplementary Figure 22. Characterization of the ZnO-coated electrospun fiber (ZF) via ALD. SEM images of the electrospun PVDF fibres from back (**a-c**) view with different coating thickness (**a**) 10 nm, (**b**) 20 nm, (**c**) 50 nm, and corresponding elemental EDX spectrum of GZF interfacial layer highlighted by yellow with elemental mapping of C, O and Zn distribution. Scale bar, 20μm for (**a-c**).

Supplementary Notes

Supplementary Notes for Supplementary Figure 16:

To better understand the role of GZCNT in suppressing dendrites, Li plating/stripping on Cu current collector with different coating layers was investigated using a half cell constructed on a Li electrode with a capacity of 3 mA h cm^{-2} at a current density of 2 mA cm^{-2} (Supplementary Fig. 16.)

During each discharge/charge cycle, a fixed amount of Li metal (3.0 mA h cm^{-2}) was plated onto the Cu electrode, and then stripped away. Thus, the Coulombic efficiency (CE) here quantifies the amount of Li metal recovered from the working electrode in the reverse stripping process, offering an important parameter to evaluate the cycling efficiency of batteries. As shown in Supplementary Fig. 16a, the GZCNT coated Cu electrodes exhibit greatly improved cycling stability with enhanced CEs. The CEs of GZCNT coated electrodes can be improved up to ~99.5% at a current density of 2.0 mA cm^{-2} with an extended cycle life of over 100 cycles. In comparison, the CEs of bare Cu electrodes start to fluctuate markedly after 30 cycles at 2.0 mA cm^{-2}, indicating the formation and sporadic activation of 'dead Li' and unstable anode/electrolyte interface during cycling.

The huge difference between GZCNT coated Cu electrodes and bare Cu electrode can also be reflected by the change of cell polarization (hysteresis) state with extended cycling. The voltage hysteresis of the Cu foil increases to above 50 mV only after 20 cycles, indicating continuous reaction between Li metal and electrolyte along with increasing internal resistance caused by the thickened SEI films. While the voltage hysteresis of the GZCNT coated Cu foil cell is maintained less than 15 mV after 100 cycles (Supplementary Fig. 16b-d), and this demonstrates its promising prospect in high energy batteries. By contrast, the ZCNT coated Cu layer, which is lithiophilic, displays lower cell polarization, while Li can be easily deposited on the surface of CNT with uniform deposition of ZnO, and then the interspace in the layer is slowly stuffed by Li deposits as the cycles continue (Supplementary Fig. 16c). Mossy Li tends to form on the stuffed ZCNT interfacial layer, leading to the failure of the cell (Supplementary Fig. 16f). The CE enhancement of ZCNT coated Cu electrode thereby was very limited compared with plain Cu, which can also be confirmed by the morphology of the deposited Li metal on these current collectors after different cycles.

According to the top view SEM images, after long-term cycling, many long Li filaments can be observed for the plain Cu current collector, and the evolution of this filament aggregated structure also leads to the uneven distribution of the electrical field, further accelerating inhomogeneous Li deposition,

which may pierce the separator and cause a safety hazard. In comparison, for a GZCNT coated Cu layer, the morphology of CNT is well retained, and no dendrite was visible. To further reveal the Li deposition process of GZCNT coated Cu layer, we show SEM images of cross-section view morphology of pristine GZCNT coated Cu foil after striping and plating back 3 mAh cm^{-2}, and after 500 cycles at 2 mA cm^{-2} with various magnifications. The GZCNT interfacial layer tightly anchored on the surface of Li deposits on Cu current collector (Supplementary Fig. 16g and h). In contrary to the porous structure on the top of GZCNT, the interfacial layer at the bottom is very dense and homogenous (Supplementary Fig. 16i). The forming of this compact interface is due to stable and uniform SEI from the lithiophilic layer, which effectively ensure the uniform Li plating/striping.

In brief, the gradient GZCNT layer was further demonstrated to be successful in regulating the deposition of Li on Cu current collector. The top lithiophobic part of GZCNT layer kept a porous morphology to facilitate the Li diffusion and hinder the dendrite forming, while the bottom lithiophilic layer anchored can effectively ensure an evenly Li plating by building a stable SEI, resulting in the fact that neither dendrites that shooting out of the electrode nor the corrosion layer between CNT and Cu foil are formed.

Supplementary Notes for Supplementary Figure 17:

The gradient strategy is not only applicable to carbon materials like CNT, but also fits well in other materials for Li anode modification. In light of this, the strategy was typically applied to a gradient ZnO/electrospun fiber (GZF) interfacial layer, which was proved equally effective in SEI stabilization and guiding the deposition of Li metal.

The GZF layer was deposited through magnetron sputtering. The ZnO gradient structure can be confirmed by cross-section SEM images and the corresponding Zn elemental EDX mapping (Supplementary Fig. 18-20). The Zn contents of the GZF layer gradually increase from top to bottom, with of 0, 8.52, and 42.43 wt %, respectively (Supplementary Fig.18-20), demonstrating the ZnO gradient loading property.

The Galvanostatic cycles of Li|Li symmetrical cells were also conducted to probe the long-term cycling stability of GZF layer-coated Li electrode. When the cells were cycled at a current density of 1.0 mA cm^{-2} with a cycling capacity of 1.0 mAh cm^{-2}, the Li|Li cell exhibited a gradually increasing hysteresis to 300 mV in less than 50 cycles, indicating an elevated charge-transfer resistance due to unstable Li/electrolyte interface and growth of dendritic Li. While the GZF-Li| GZF-Li cell displays an excellent cycling stability as evidenced by a negligible voltage fluctuation and a much lowered overpotential (~80 mV in the initial cycles, and further stabilizes at 45 mV for more than 500 cycles (~1000 h)) (Supplementary Fig. 17c). During the cycling process, a quick decrease of the hysteresis indicates a fast SEI formation, after cycling for over 1000 h, no sign of voltage oscillations is observed, confirming an effective suppression of dendrite growth. Similar tests were carried out under higher current densities of 5 mA cm^{-2}, the GZF-Li|GZF-Li cell, with a corresponding overpotential of 100 mV (Supplementary Fig. 17d), can still run for 135 h. These results suggest that a stable and homogeneous SEI forms between the GZF interfacial layer and Li, which ensures enhanced charge-transfer kinetics. The electrochemical performance of the cells assembled by Li foils with uniform ZnO/electrospun fiber (ZF) by atomic layer deposition (ALD) is compared with that of the bare Li foils (Supplementary Fig. 21-22). The ZF–Li and Fiber–Li cell all display a large voltage fluctuation and their voltage quickly exceed 2 V.

The highly reduced polarization and stable cycling of GZF interfacial layer can be further supported by the electrochemical impedance spectroscopy (EIS) conducted on symmetric as prepared cells and after 130 cycles. The corresponding Nyquist plots are shown in Supplementary Fig. 17b. It is noteworthy that the interfacial ionic resistance in the SEI layer (referring to the semicircle in high

frequency of EIS) reduce with the prolonged cycles in the GZF coated Li cell. This indicates that the bottom lithiophilic layer may effectively ensure an evenly Li plating and a stable SEI layer by regulating deposition. The interfacial ionic resistances of the bare Li foil cells increase with prolonged cycles, due to the "dead Li" layer with more SEI formation and unstable surface structure on Li foil (Supplementary Fig. 17b). After 130 cycles, the R_{SEI} of the plain Li foil cell increased from 220 to 275 Ω while the GZF coated cell decreased from 230 to 75 Ω (Supplementary Fig. 17b). This result demonstrates that a good and stableLi stripping/plating kinetics can be achieved with the GZF coated Li electrode.

The difference between ZF and GZF can also be elucidated by the SEM images of electrodes after 520 cycles. It is obvious that some large Li lumps are deposited unevenly on electrospun fiber (Supplementary Fig. 17e), most of the electrospun fiber layer is buried underneath the Li deposits after 220 cycles, which is attributed to its flexible and soft properties that can be easily bent and coated by newly grown Li deposits. For the ZF layer coated Li foil, which is lithiophilic, Li can be easily deposited on the surface of ZnO coated electrospun fibers (Supplementary Fig. 17f), as the cycles continue, the layer will be slowly stuffed by Li deposits, finally, mossy Li tends to form on the stuffed ZF interfacial layer, leading to the failure of the cell. By contrast, no obvious crevice or dendrite is visible for GZF layer (Supplementary Fig. 17g). The GZF layer is tightly anchored on Li foil, and a stable SEI is formed between them (Supplementary Fig. 17h), which confirms the significance of gradient layer strategy in lithium dendrite suppression.

In brief, this gradient strategy was further validated efficient in electrospun fiber, which can improve cycle performance and stand out from the carbon-based interfacial layer approach for Li anode modification previously reported.
